# New Heteroleptic Ruthenium(II) Complexes with Sulfamethoxypyridazine and Diimines as Potential Antitumor Agents

**DOI:** 10.3390/molecules24112154

**Published:** 2019-06-07

**Authors:** Ariane C.C. de Melo, Jaime M.S.V.P. Santana, Kelen J.R.C. Nunes, Bernardo L. Rodrigues, Nathalia Castilho, Philipe Gabriel, Adolfo H. Moraes, Mayra de A. Marques, Guilherme A.P. de Oliveira, Ívina P. de Souza, Hernán Terenzi, Elene C. Pereira-Maia

**Affiliations:** 1 Department of Chemistry, Universidade Federal de Minas Gerais, Belo Horizonte 31270-901, MG, Brazil; ariane.quimica@hotmail.com (A.C.C.d.M.); jaimemurilosvps@gmail.com (J.M.S.V.P.S.); cdkelen@hotmail.com (K.J.R.C.N.); bernardo@qui.ufmg.br (B.L.R.); adolfo.dq.ufmg@gmail.com (A.H.M.); ivina_paula@yahoo.com.br (Í.P.d.S.); 2 Department of Biochemistry, Universidade Federal de Santa Catarina, Florianópolis 88040900, SC, Brazil; nathi_zuca@hotmail.com (N.C.); philipe.gabriel.ph@gmail.com (P.G.); hterenzi@ccb.ufsc.br (H.T.); 3 Programa de Biologia Estrutural, Instituto de Bioquímica Médica Leopoldo de Meis, Instituto Nacional de Biologia Estrutural e Bioimagem, Centro Nacional de Ressonância Magnética Nuclear Jiri Jonas, Universidade Federal do Rio de Janeiro, Rio de Janeiro 21941590, RJ, Brazil; mayra.marques@ymail.com (M.d.A.M.); gaugusto@bioqmed.ufrj.br (G.A.P.d.O.); 4 Department of Chemistry, Centro Federal de Educação Tecnológica de Minas Gerais, Belo Horizonte 30421-169, MG, Brazil

**Keywords:** ruthenium complexes, sulfonamide, Abl tyrosine kinase, bovine serum albumin, DNA

## Abstract

Two new complexes of Ru(II) with mixed ligands were prepared: [Ru(bpy)_2_smp](PF_6_) (**1**) and [Ru(phen)_2_smp](PF_6_) (**2**), in which smp = sulfamethoxypyridazine; bpy = 2,2′-bipyridine; phen = 1,10-phenanthroline. The complexes have been characterized by elemental and conductivity analyses; infrared, NMR, and electrospray ionization mass spectroscopies; and X-ray diffraction of single crystal. Structural analyses reveal a distorted octahedral geometry around Ru(II) that is bound to two bpy (in **1**) or two phen (in **2**) via their two heterocyclic nitrogens and to two nitrogen atoms from sulfamethoxypyridazine—one of the methoxypyridazine ring and the sulfonamidic nitrogen, which is deprotonated. Both complexes inhibit the growth of chronic myelogenous leukemia cells. The interaction of the complexes with bovine serum albumin and DNA is described. DNA footprinting using an oligonucleotide as substrate showed the complexes’ preference for thymine base rich sites. It is worth notifying that the complexes interact with the Src homology SH3 domain of the Abl tyrosine kinase protein. Abl protein is involved in signal transduction and implicated in the development of chronic myelogenous leukemia. Nuclear magnetic resonance (NMR) studies of the interaction of complex **2** with the Abl-SH3 domain showed that the most affected residues were T79, G97, W99, and Y115.

## 1. Introduction

The discovery of the cytotoxic effects of *cis*-diamminedichloroplatinum(II), or cisplatin, in the 1960s, by Rosenberg et al. prompted the search for other antitumoral metal compounds that could be used to treat cancer [1]. The antitumor action of cisplatin is mainly associated to the formation of cisplatin-DNA adducts [2,3]. Several coordination compounds possessing antitumoral properties are now described in the literature as well as different strategies to improve the activity of known agents such as the preparation of new delivery systems and the conjugation of drugs to proteins [4].

A variety of ruthenium complexes have been designed aiming at the treatment of cancer [5,6,7,8,9]. Three compounds of ruthenium(III), the imidazolium *trans*-tetrachloro(dimethylsulfoxide)(1H-imidazole)ruthenate(III) or NAMI-A, its sodium analogue or IT-139, and the indazolium *trans*-tetrachlorobis(1H-indazole)ruthenate(III) or KP1019, entered clinical trials. In spite of their structural and chemical similarities, KP1019 and NAMI-A show distinct antitumor patterns: NAMI-A is more effective against cancer metastases than against primary tumors, whereas the activity of KP1019 is predominantly due more to direct cytotoxic effects than to an interference with the process of tumor cell invasion and metastasis [10,11,12,13,14].

Ruthenium(II) complexes containing N-heterocyclic ligands and organometallic Ru(II) arene complexes containing different chelating ligants were also reported to exhibit anticancer activity [15,16]. The mechanism of action of ruthenium compounds is not yet fully understood and both DNA and protein binding were reported [17,18,19,20]. Ru(II) metallopeptides containing the dipyridophenazine ligand functionalized with octaarginine domains selectively bind to DNA G-quadruplex structures and are efficiently internalized, causing cell death by apoptosis [21]. 

Ruthenium complexes are highly cytotoxic in leukemia cells, a cancer of early blood-forming cells [22,23]. In chronic myelogenous leukemia (CML), a genetic change leads to the fusion of the *bcr* and *abl* genes, which together encode for a cytoplasm-targeted deregulated form of Abl tyrosine kinase, which is responsible for the development of CML. The activity of Abl kinase is negatively regulated by the Src homology domains 2 and 3, SH2 and SH3, clamping the protein in an inactive state [24,25]. Although the development of Abl inhibitors, such as the imatinib, nilotinib, or dasatinib, has improved the clinical management of CML, the evolution of drug-resistant mutants makes the search for newer classes of Abl inhibitors an urgent task [26].

Polypyridyl ruthenium complexes are also receiving great attention due to their potential in photodynamic therapy, i.e., an emerging anticancer treatment that comprises the presence of light, oxygen, and a photosensitizing drug to accomplish the photocytotoxic effect [27,28,29,30,31,32,33]. The photoactivated drug produces reactive oxygen species that can oxidize important cellular components such as DNA and cause cell death. Oligonucleotide-conjugates containing [Ru(phen)_3_]^2+^ as photosensitizer groups induced photooxidative damage on single-stranded DNA representing the bcr-abl chimeric gene [34]. 

**Figure 1:**
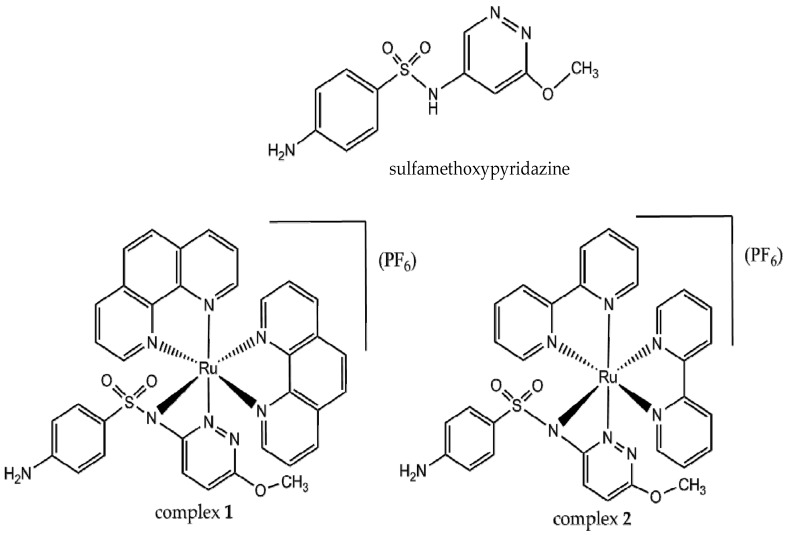
Chemical structures of sulfamethoxypyridazine and the synthesized complexes.

Coordination of active metal ions to antibiotic molecules can improve the pharmacological activity [35]. Sulfonamides, the first synthetic antibiotics to be used in clinic, exhibit interesting pharmacological properties such as low toxicity. Sulfamethoxypyridazine, shown in Figure 1, is useful in the treatment of vaginal irritation, urinary infections, thrush, and acute dermatitis herpetiformis [36]. Sulfonamides are able to form complexes with various metal ions [37,38,39,40,41,42]. In a previous work, we described a complex of Bi(III) in which bismuth is bound to three sulfapyridine molecules through sulfonamidic nitrogens and to three chloride ions. The compound inhibited the growth of K562 cells while free sulfapyridine is not active [42]. More recently, Refat et al. prepared Ru(III) complexes of type ML_2_, in which L = sulfamethoxazole, sulfanilamide, sulfadimidine, or sulfadiazine and suggested that coordination occurs through the aniline and sulfonamidic nitrogens [43].

The described pharmacological properties of sulfonamides and those of ruthenium complexes stimulated us to study the antitumoral potential of ternary complexes of ruthenium(II) containing a sulfonamide and a N-donor heterocyclic as ligands. This work reports on the synthesis and characterization of two complexes of ruthenium(II) with sulfamethoxypyridazine (smp) and either 1,10-phenanthroline (phen) or 2,2′-bipyridine (bpy) as ligands (Figure 1). Their cytotoxic effect and interactions with DNA, bovine serum albumin (BSA), and the SH3 domain of the Abl tyrosine kinase protein were also studied. 

## 2. Results

### 2.1. Characterization of Complexes ***1*** and ***2***

**Figure 2:**
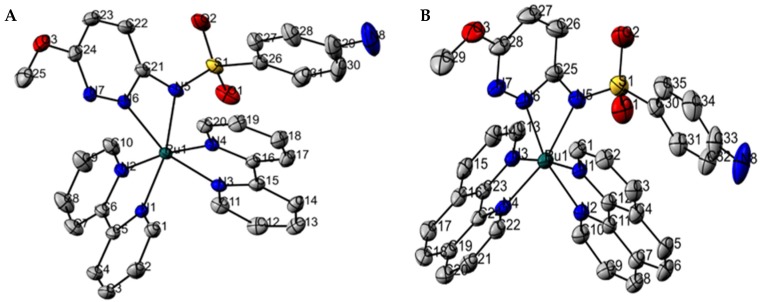
ORTEP projection of the molecular structure of complexes **1** (**A**) and **2** (**B**) with the symbology of the atoms involved in the coordination. Non-hydrogen atoms are represented as 50% probability ellipsoids. Hydrogen atoms are omitted for clarity. In complex **1**, the bond angles N(6)–Ru(1)–N(5), N(3)–Ru(1)–N(4), N(1)–Ru(1)–N(2) are 62.11(7)°, 79.23(7)°, and 79.22(7)°, respectively, whereas in complex **2**, N(6)–Ru(1)–N(5), N(3)–Ru(1)–N(4), N(1)–Ru(1)–N(2) are 62.20°(12), 79.88°(10), 80.18°(10), respectively. The bond lengths Ru(1)–N(1), Ru(1)–N(2), Ru(1)–N(3), Ru(1)–N(4), Ru(1)–N(5), Ru(1)–N(6) are 2.0451 (16), 2.0514 (16), 2.0392 (18), 2.0482 (17), 2.1080 (17), 2.0982 (18) Å, respectively for complex **1**. The Ru-N bond lengths for complex **2**: Ru(1)–N(1), Ru(1)–N(2), Ru(1)–N(3), Ru(1)–N(4), Ru(1)–N(5), Ru(1)–N(6), 2.062 (3), 2.066 (3), 2.059 (3), 2.044 (3), 2.131 (3), 2.062 (3).

The details concerning data collection and structure refinement are given in the supplementary Table S1. The ORTEP diagrams are shown in Figure 2. The crystal structures of both complexes show similar geometry and coordination environment around the ruthenium atom. The coordination sphere is composed by two nitrogen atoms from two molecules of bpy or phen (N1, N2, N3, and N4) and two nitrogen atoms from sulfamethoxypyridazine: One of the methoxypyridazine ring (N6) and the sulfonamidic nitrogen (N5), which is deprotonated. In both cases, the ruthenium atom adopts a slightly distorted octahedral geometry.

The most relevant bond lengths and bond angles in **1** and **2** are listed in Table S2. The Ru-N bond lengths are in the range reported in previous studies [7,20,23,31]. The bond lengths Ru(1)-N(5) and Ru(1)-N(6) are longer than the others due to the coordination mode of smp, which forms a four-membered chelate ring with Ru(II) atom through N(5) and N(6). A three-dimensional network of hydrogen bonds {O–H∙∙∙O, N–H∙∙∙O, N–H∙∙∙N, and O–H∙∙∙N} contributes to the stabilization of complex **1** (Figure S1). A water molecule is alternately arranged between the asymmetric units of the complex in crystal lattice, participating in intermolecular hydrogen bonds with bpy and smp (through the N of the bpy ring and N of the sulfonamide group). In addition, the intercentroid distance between bpy and smp rings, 3.739 Å, indicates the presence of π–π parallel-displaced interactions between aromatic bpy rings from different complex **1** cations.

In complex **2**, a molecule of isopropyl alcohol is involved in classical hydrogen bonds that contribute to the stabilization of the crystalline network of the compound. As in complex **1**, parallel-displaced π–π interactions occur between phen and smp rings besides to face-to-face π–π interactions between distinct phen rings (Figure S2). The distance between the smp and phen centroids is 3.657 Å, i.e., a value greater than the distance between the centroids of two phen ligands whose value is about 3.392 Å. Geometric parameters of the hydrogen bonds present in **1** and **2** are shown in Table S3.

The infrared spectra of both complexes in comparison to that of the corresponding free sulfa ligand are in accordance with the coordination via the sulfonamidic nitrogen. The absorption at 3162 cm^−1^, assigned to the ν(N–H) of smp was not observed in the spectra of the complexes, attesting its deprotonation. The band attributed to the stretching ν(N=N), observed at 998 cm^−1^ on the sulfamethoxypiridazine spectrum, shifts by 14–26 cm^−1^ in the complexes (Figures S3 and S4). 

The conductivity values of 10^−3^ mol L^−1^ solutions of compounds **1** and **2** in nitromethane are in the range of 1:1 electrolyte (75–95 ohm^−1^ cm ^2^ mol^−1^) [44]. 

**Figure 3:**
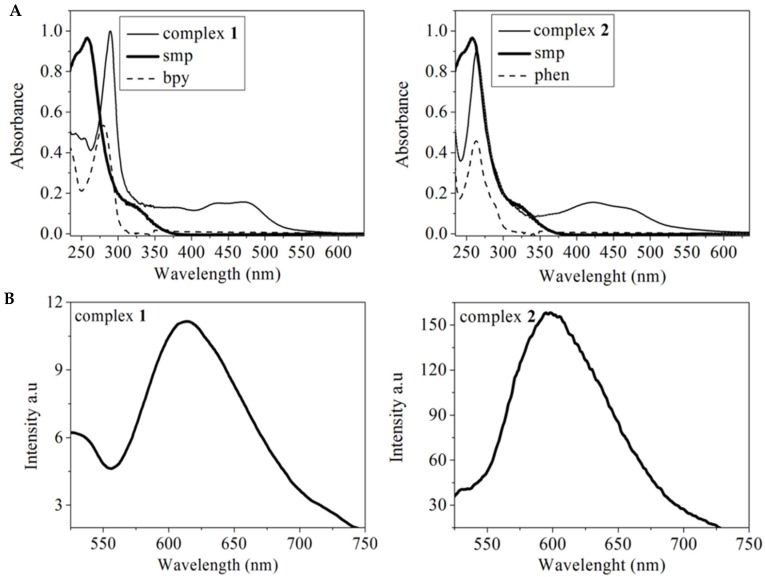
Electronic spectra of aqueous solutions of complexes **1** and **2** at 3.0 × 10^−5^ mol L^−1^ (**A**). Fluorescence emission spectra of complex **1** at 5.0 × 10^−4^ mol L^−1^ **1** and complex **2** at 5.0 × 10^−5^ mol L^−1^, λexc = 472 nm (**B**).

The electronic spectra of complexes **1** and **2** with their respective ligands are shown in Figure 3A. The ligands bpy, phen, and smp exhibit bands centered at 280 nm, 264 nm, and 257 nm, respectively, due to intraligand π–π* transitions, which undergo bathochromic shifts in the complexes. New bands in the region of 400–500 nm appear in the spectra of complexes due to metal-to-ligand charge transfer transitions (MLCT, d–π) [45]. The UV-vis spectra did not change with time, in up to 24 h, attesting to the complexes’ inertness in aqueous solution. The fluorescence spectra of complexes **1** and **2** show one emission band centered at 613 and 597 nm, respectively, when excited at 472 nm (Figure 3B).

In the ^1^H NMR spectra of complex **1** and **2**, the methoxy and amine protons from the smp ligand give rise to signals at δ 3.61, 5.68, and δ 3.54, 5.55, respectively. In the range δ 6−9.8 the signals of aromatic protons from smp appear overlapped with those from the N-heterocyclic ligand (bpy or phen). The main modification observed in the ^1^H NMR spectra of the complexes in relation to that of the free ligand is the absence of a resonance at δ 11.90 assigned to the proton of the sulfonamidic nitrogen, indicating its deprotonation. 

The presence of the complexes in solution was confirmed by ESI-MS studies. The ESI-MS spectrum of complex **1** in positive mode gives a main peak at *m/z* 693.09 assigned to [Ru(C_10_H_8_N_2_)_2_C_11_H_11_N_4_O_3_S]^+^ (Figure S5) and that of complex **2** at *m/z* 741.09 assigned to the species [Ru(C_12_H_8_N_2_)_2_C_11_H_11_N_4_O_3_S]^+^ (Figure S6). The isotopic distribution for the proposed species was calculated with the program LabSolutions/LCMSSolutions (2010) and there is a good accordance with the experimental spectra.

### 2.2. Cytotoxic Effect on Myelogenous Leukemia Cells

Both complexes were able to inhibit the growth of myelogenous leukemia cells in a concentration dependent manner. The IC_50_ values obtained for complexes **1**, **2** and their respective ruthenium precursors are indicated in Table 1. The replacement of chlorides by sulfamethoxypyridazine confers cytotoxic activity to the complexes as either *cis*-[RuCl_2_(bpy)_2_] or *cis*-[RuCl_2_(phen)_2_] are inactive. By comparing the effect of the 1,10-phenanthroline to that of 2,2’-bipyrydine one can infer that the former strengths further the cytotoxicity. 

### 2.3. DNA Binding and Photocleavage

Firstly, the effect of compounds in the dark was investigated by incubating plasmid DNA with increasing complex concentrations from 5 to 500 μM, for 16 h, at 37 °C. There is no cleavage without light exposure.

**Figure 4:**
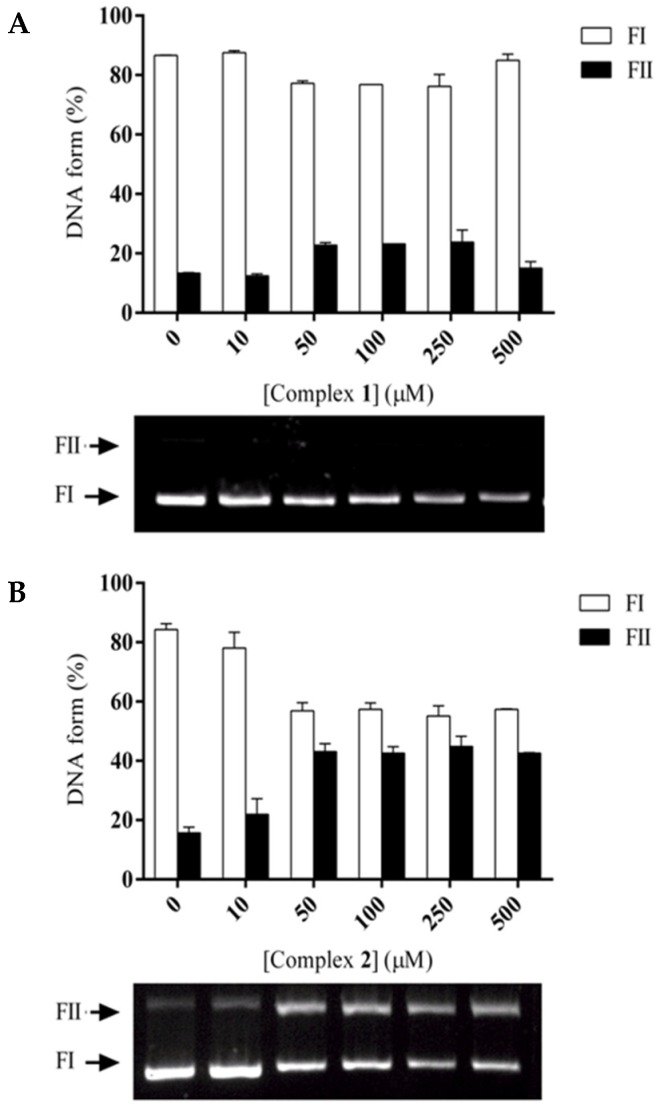
Photocleavage of supercoiled DNA by **1** (**A**) and **2** (**B**) (after 10 min of UV-A exposure) at 37 °C, pH 7.0.

Afterwards, the effect of UV-light in DNA cleavage was studied at different irradiation times. Figure 4 shows the cleavage of supercoiled form of plasmid DNA by different concentrations of complexes **1** and **2** after 10 min of light exposure. DNA cleavage is clearly concentration dependent with a similar pattern for both complexes. Complex **1** at the concentration of 50 µM converts about 25% of supercoiled form (FI) of plasmid DNA to its open circular form (FII). Complex **2** is more active converting approximately 45% of supercoiled DNA to the open circular form in the same conditions. The absence of the linear form (FIII) suggests that the complexes only induce single-strand scission in DNA. From 100 µM, the events of cleavage reach saturation. After 15 min of UV light exposure, complex **1** causes about 45% of DNA cleavage whereas complex **2** about 70% of DNA cleavage (Figures S7 and S8).

In order to evaluate the kinetic profile of the complexes **1** and **2**, they were incubated at different concentrations and times. Assuming that we have as substrate the plasmid DNA and as catalyst the metal complexes, we considered that the curve generated by these data assumes the conformation of a *pseudo*-Michaelis-Menten, where the variable is the concentration of catalyst (Figure S9). Complex **1**, displays a *k_cat_* (cleavage rate constant) of 1.38 h^−1^, while **2** presented the value of 2.03 h^−1^ (Table 2).

When the other parameters were analyzed (K_M_ and *k_cat_*/K_M_), complex **2** presented kinetic constants compatible with higher activity and affinity when compared with complex **1**. We also observed this effect in metal complexes with Cu(II) when photoactivated [46]. 

**Figure 5:**
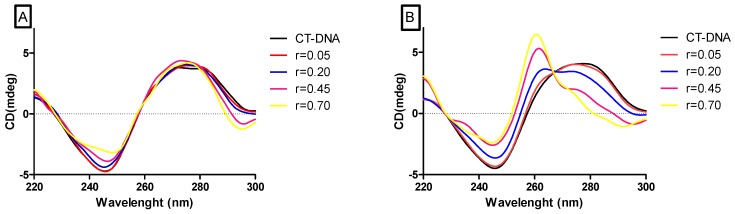
Circular dichroism spectra of CT-DNA (200µM) in the absence or presence of varying concentrations of the complexes 1 (**A**) and 2 (**B**). (r = 0.05, r = 0.20, r = 0.45, r = 0.70) where r = [complex]/[CT-DNA].

When the circular dichroism (CD) spectra (Figure 5) were analyzed, both complexes bind to DNA and modify its secondary structure profile, which is evidenced by CD signals alterations in the presence of increasing concentrations of each complex. As exposed in Figure 4B, complex **2** interacts with DNA causing larger modifications in the secondary structure when compared to complex **1** (Figure 4A).

The cleavage by **1** and **2** is not modified with pH variation (Figure S10). These results may indicate an oxidative type of cleavage, where the protonation/deprotonation profile associated in general with hydrolytic mechanism is not observed. In the oxidative mechanism, in opposition to hydrolytic mechanism, the deprotonation of water molecules for the nucleophilic attack does not occur, thus the modification in the pH values would not interfere in the cleavage of plasmid DNA, as analyzed in the experiment.

In order to evaluate the possible preference of DNA binding by complexes **1** and **2**, we performed DNA hydroxyl radical footprinting assays (see methods section) using an oligonucleotide as substrate. The oligonucleotide presents 21bp and some peculiar characteristics: AT and CG rich regions and two single-stranded structures composed of thymine bases. These characteristics provide different information on how the complexes’ interaction with DNA may be occurring. As can be clearly seen, the DNA cleavage promoted by the oxidizing agent decreased in the presence of both complexes as shown in Figure S10, creating “footprints” in the sequence that may be mapped to the DNA structure. Mapping the oligonucleotide sequence protected by both complexes, and identified as A, B, and C in Figure S10, three binding preference regions were identified that are rich in thymine bases and preferentially single-strand structures.

### 2.4. BSA Binding Studies

The knowledge of the binding affinity of a compound to serum albumin is important to estimate its usefulness as a therapeutic agent. The effective dose levels for a drug rely on the level of unbound drug in the circulation because binding to serum albumin prevents the binding to the pharmacological target.

**Figure 6:**
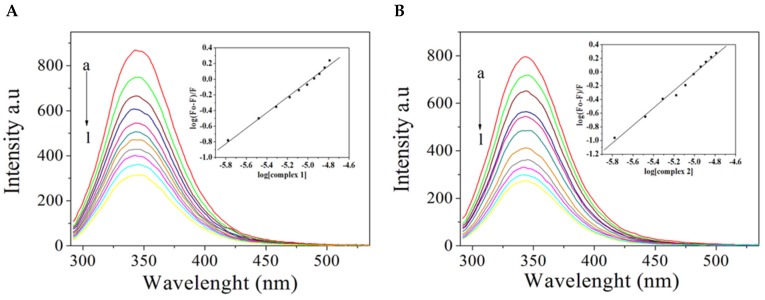
Interaction of complexes **1** (**A**) and **2** (**B**) with bovine serum albumin. Fluorescence emission spectra of BSA (1.0 × 10^−6^ mol L^−1^, excitation = 280 nm) in the presence of increasing complex concentrations. Complex-to-BSA molar ratios are a = 0, b = 1.66, c = 3.31, d = 4.95, e = 6.57, f = 8.19, g = 9.80, h = 11.40, i = 12.90, j = 14.50, l = 16.10. Inset: log (F_0_-F)/F] versus log [Q].

The compounds quench the fluorescence emission of BSA, centered at 340 nm, when excited at 280 nm, indicating an interaction with the protein (Figure 6). Fluorescence data were analyzed with the help of the Stern–Volmer equation, and the Ksv and the binding constant (Kb) values obtained are listed in Table 3. In both cases, the number of binding sites is approximately one, which indicates that there is only one binding site in BSA for the studied complexes. Complex **1** has a lower affinity to BSA than complex **2**. These values are comparable to those reported for other ruthenium complexes with BSA [47,48,49]. The binding constant of a compound to serum albumin should be high enough to guarantee its transportation and distribution but low enough to ensure that the compound will be released to reach its pharmacological target [50]. The binding constants of complex **1** and **2** are within such an optimum range, 8.19 × 10^4^ and 2.47 × 10^6^.

### 2.5. Interaction with the ABL-SH3 Domain

**Figure 7:**
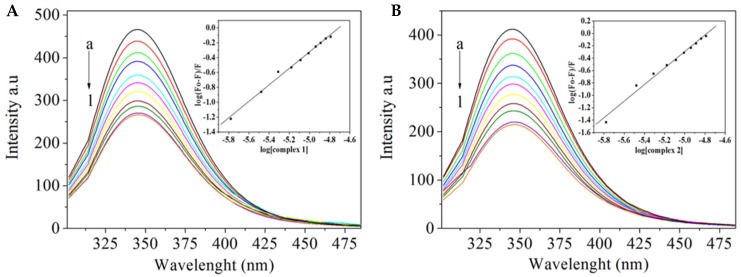
Interaction of complexes **1** (**A**) and **2** (**B**) with Abl-SH3. Fluorescence emission spectra of Abl-SH3 (1.0 × 10^−6^ M, excitation = 280 nm) in the presence of increasing complex concentrations. Complex-to-Abl-SH3 molar ratios are a = 0, b = 1.66, c = 3.31, d = 4.95, e=6.57, f = 8.19, g = 9.80, h = 11.40, i = 12.90, j = 14.50, l = 16.10. Inset: log (F_0_-F)/F] versus log [Q].

The interaction of complexes **1** and **2** with Abl-SH3 was studied by fluorescence spectrometry. The binding constants determined for complexes **1** and **2** were, respectively, 1.70 × 10^5^ and 2.50 × 10^6^ L mol^−1^. Representative titrations of Abl-SH3 with the complexes can be seen in Figure 7.

**Figure 8:**
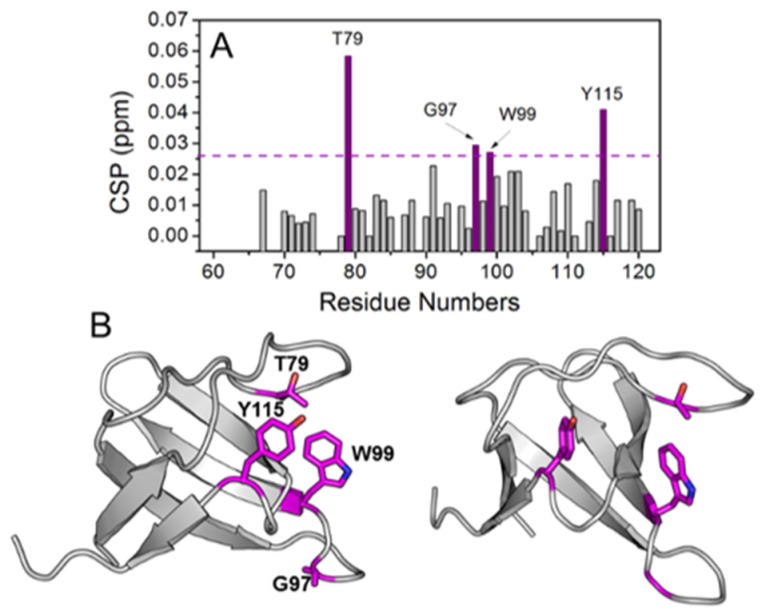
(**A**) Chemical Shift Perturbation index (CSP) as a function of ABL-SH3 primary sequence. CSP values were calculated from the difference of ^1^H and ^15^N chemical shift values on ^1^H–^15^N sofast-HMQC spectra of ^15^N-labeled ABL-SH3 free and in solution with complex **2**. The cutoff line, colored in magenta, is the sum of the mean value and the standard deviation, both calculated using all CSP values. (**B**) Residues with CSP higher than the cutoff value were colored in magenta on the crystal structure of ABL-SH3 (PDB ID: 4JJC). The side chains of those residues are also highlighted.

The interaction of complex **2**, which is the most active, with the Abl-SH3 domain was also investigated by NMR spectrometry, Figure 8A. The interaction was monitored using the chemical shift perturbation (CSP) observed on the ^1^H–^15^H heteronuclear correlation spectrum of ^15^N-labeled Abl-SH3. Four residues showed CSP higher than the average plus the standard deviation of all CSP’s: T79, G97, W99, and Y115. Those residues are dispersed in the Abl-SH3 primary sequence, but when they are colored in magenta and their side-chains shown in the Abl-SH3 structure (Figure 8B), a conformation-binding site formed by their side-chains is easily visualized.

Src-family kinases (SFKs), as Abl tyrosine kinase, mediate important biology invents in human biology, being involved in both upstream and downstream interactions. Because of that, SFKs could be key drug targets against many diseases [51]. SFK-SH3 domain is a potential powerful new target if truly selective inhibitors can be developed. The development selective inhibitors of SH3 is hampered by the high primary sequence conservation among SH3 domains from different SFKs. Therefore, many efforts focusing the discovery of new high specific drugs interacting with SH3 domain are being done. Recently, Vohidov, F et al. characterized rhodium(II) conjugates, which increase considerably the inhibition of Lyn, Lkc, and Abl Src-family SH3 domain by specific peptides formulated from different protein partners [52]. They showed that the addition of the rhodium(II) conjugate center increased by many folds the affinity of peptides to SH3. Specifically for Abl-SH3, the metallopeptide produced from P40 peptide with the addition of a rhodium(II) center decreases the dissociation constant, Kd, from 400 to 22 nM [52].

Our results represent one of the first studies focusing the interaction of Abl-SH3 with a ruthenium complex, which could be combined with other compounds, such as peptides and peptoids to formulate more efficient Abl-SH3 inhibitors.

## 3. Materials and Methods

### 3.1. General and Instruments

4-Amino-N-(6-methoxypyridazin-3-yl)benzenesulfonamide (smp), 2,2′-bipyridine (bpy), 1,10-phenathroline (phen), and RuCl_3_ were purchased from Sigma Co. (St. Louis, MO, USA). All other chemicals were reagent-grade and were used without further purification.

Elemental analyses were performed on a Perkin–Elmer 2400 CHN analyzer (Waltham, MA, USA). Conductivity studies were carried out with a Digimed DM 31 conductivity meter using a cell of constant 1.130 cm^−1^, spectroscopic grade nitromethane (Merck) (ΛM = 8.20 S cm^2^ mol^−1^) and tetramethylammonium bromide (ΛM = 79.12S cm^2^ mol^−1^) as a standard. Infrared spectra were recorded over the region 400–4000 cm^−1^ with a Perkin–Elmer 283 B spectrometer, (Waltham, MA, USA). The samples were examined in KBr pellets. A Cary 100 Varian spectrometer (Santa Clara, CA, USA) was used for UV and visible absorption measurements. Fluorescence spectra were recorded in a Shimadzu RF5301PC spectrophotometer (Kyoto, Japan). A stock solution of the complexes (1 × 10^−2^ M) was prepared in acetonitrile and further diluted in HEPES buffer (20 mM), pH 7.2. Circular dichroism spectra were recorded in a spectropolarimeter J-815 (JASCO, Easton, MD, USA). NMR spectra were obtained in a Bruker Avance DRX 400 spectrometer (Billerica, MA, USA) with tetramethylsilane as an internal standard using dmso-*d6*. Full scan mass spectra were obtained in a MicroTOF LC Bruker Daltonics spectrometer (Fremont, CA, USA), equipped with an electrospray source operating in positive ion mode. Samples were dissolved in acetone/acetonitrile (1/1) and were injected in the apparatus by direct infusion.

### 3.2. Synthesis of Complexes [Ru(bpy)_2_smp](PF_6_) and [Ru(phen)_2_smp](PF_6_)

The precursors, *cis*-Ru(bpy)_2_Cl_2_ and *cis*-Ru(phen)_2_Cl_2_, were prepared according to a literature method [44]. Complexes **1** and **2** were prepared using a general procedure, in which the precursor (0.40 mmol, 0.21 g of *cis*-Ru(bpy)_2_Cl_2_ or 0.40 mmol, 0.23 g of *cis*-Ru(phen)_2_Cl_2_) and smp (0.60 mmol, 0.168 g) were mixed in 40.0 mL of an ethylene glycol/water solution (7:1, *v*/*v*). The reaction mixture was refluxed for 6 h, while the solution turned from purple to red. Afterwards, 40.0 mL of water was added and the mixture was filtered to remove solid impurities. Subsequently 12.27 mmol (2.31 g) of KPF_6_ was added to the filtrate, which leads to the formation of a dark red solid. The complexes were separated by filtration, washed thoroughly with water, ethanol, and diethyl ether and dried. Furthermore, the complex was purified by alumina chromatography, using acetone/ethyl acetate (1:10, *v*/*v*) as eluent. Crystals suitable for single crystal X-ray diffraction were obtained by slow evaporation from a 1:1 acetonitrile/isopropyl alcohol solution.

Complex **1**: Yield 70.2%. IR (KBr): νmax = 3478, 3382, 1596, 1328, 1160, 1024, 974, 842 cm^−1^. ΛM= 93.79 S cm^2^ mol^−1^ in nitromethane. Electronic spectrum (0.3% acetonitrile in water pH 7.2) λmax = 472 nm, ε = 5233 L mol^−1^ cm^−1^). ^1^H NMR (dmso-*d6*) δ 6.13–9.20 (24H, m) δ 5.68 (2H, s) δ 3.34 (3H, s). Anal. calc. for [Ru(C_10_H_8_N_2_)_2_C_11_H_11_N_4_O_3_S](PF_6_) ∙ 0.4 H_2_O (845.49 g mol^−1^): C 44.07, H 3.32, N 13.26, found C 43.64, H 3.64, N 13.43. ESI-MS (CH_3_CN-C_3_H_6_O 1:1) calc for [Ru(C_10_H_8_N_2_)_2_C_11_H_11_N_4_O_3_S]^+^: *m*/*z* = 693.098; found: *m*/*z* = 693.094.

Complex **2**: Yield 59.2%. IR (KBr): νmax = 3478, 3392, 1596, 1328, 1164, 1012, 972, 840 cm^−1^. ΛM = 94.28 S cm^2^ mol^−1^ in nitromethane. Electronic spectrum (0.3% acetonitrile in water pH 7.2) λmax = 470 nm, ε = 4200 L mol^−1^ cm^−1^). ^1^H NMR (dmso-*d6*) δ 6.09–9.78 (22H, m) δ 3.54 (3H, s) δ 5.55 (2H, s) δ 3.85 (1H, s) δ 2.16 (1H, s) δ 1.01 (6H, s). Anal. calc. for [Ru(C_12_H_8_N_2_)_2_C_11_H_11_N_4_O_3_S](PF_6_) ∙ C_3_H_8_O (945.83 g mol^−1^): C 48.25, H 3.73, N 11.85, found C 48.33, H 3.52, N 11.89. ESI-MS (CH_3_CN-C_3_H_6_O 1:1) calc for [Ru(C_12_H_8_N_2_)_2_C_11_H_11_N_4_O_3_S]^+^: *m*/*z* = 741.097; found: *m*/*z* = 741.093. 

### 3.3. Crystal Structure Determination

Single-crystal X-ray diffraction data were collected at 120 K on an Oxford Gemini Atlas Ultra diffractometer with graphite-monochromated, λ (MoKα) = 0.71073 Å using the CrysAlis-Pro data collection and data processing software [53]. The structures were solved using the SHELXS-97 program [54] and refined using SHELXL-14/7 [55]. The programs MERCURY [56] and ORTEP-3 [57] were used within the WinGX [58] software package to prepare artwork representation. H atoms were refined with isotropic thermal parameters. Non-hydrogen atoms were refined with anisotropic thermal parameters and were located from difference Fourier map. Correction for absorption of reflection intensities was applied. 

Complex **2** shows in its unit cell one molecule of isopropyl alcohol, which exhibits site occupancy disorder and was refined by splitting carbon and hydrogen atoms over two positions.

X-ray crystallographic data: CCDC reference numbers 1531933 and 1531934 contain the supplementary crystallographic data for complex 1 and 2, respectively. 

### 3.4. Cells, Culture and Drug Sensitivity Assays

The K562 cell line was purchased from the Rio de Janeiro Cell Bank (number CR083 of the RJCB collection). This cell line was established from pleural effusion of a 53-year-old female with chronic myelogenous leukemia in terminal blast crisis. Cells were cultured in RPMI 1640 (Sigma Chemical Co., St Louis, MO, USA) medium supplemented with 10% fetal calf serum (CULTILAB, São Paulo, Brazil) at 37 °C in a humidified 5% CO_2_ atmosphere. Cultures grow exponentially from 10^5^ cells mL^−1^ to about 8 × 10^5^ cells mL^−1^ in three days. The cell number was determined by Coulter counter analysis. 

For cytotoxicity assessment, 1 × 10^5^ cells mL^−1^ were cultured for 4 h in the absence and the presence of a range of concentrations of tested compounds. Subsequently, cells were washed and cultured in RPMI 1640 medium supplemented with 10% fetal calf serum, for 72 h. The percentage of cell growth inhibition was plotted against the concentration of compound added to the medium, and the concentration that inhibits cell growth by 50%, the IC_50_ value, was determined. Cell viability was checked by Trypan Blue exclusion. Stock solutions of the compounds were prepared in acetonitrile. The final content of acetonitrile in the culture medium was inferior to 0.3%.

### 3.5. DNA Cleavage Assays

The DNA cleavage mediated by complexes **1** and **2** was evaluated using agarose gel electrophoresis to separate the cleavage products from plasmid DNA pBSK II [59,60]. In general, 330 ng of plasmid DNA (~25 μM in base pairs) in HEPES buffer (10 mM, pH 7.0) were treated with different concentrations of complexes 1 and 2 at 37 °C with or without UV-light irradiation (λ = 365 nm, 12 W) for 5, 10, or 15 min. All assays were conducted including a reaction control (without complex) to serve as a reference of spontaneous plasmid DNA fragmentation. Thereafter, each reaction was quenched by adding 5 μL of a loading buffer solution (0.01% bromophenol blue, 50% glycerol and 250 mM EDTA at pH 7.5) and then subjected to electrophoresis on a 1% agarose gel containing 0.3 μg mL^−1^ of ethidium bromide in 0.5 × Tris/borate/EDTA (TBE) buffer (44.5 mM Tris, 44.5 mM boric acid and 1 mM EDTA at pH 8.0) at 90 V for 100 min. The resulting gels were visualized and digitized using a DigiDoc-It gel documentation system (UVP). The proportion of plasmid DNA in each band was quantified using Kodak Molecular Imaging Software 5.0 (Carestream Health, Rochester, NY, USA).

Cleavage kinetics assays were performed using a final volume of 120 μL to which 2 μg of plasmid DNA in 10 mM HEPES pH 7.0 were added, followed by 30 μL of complex at different concentrations (0–50 μM). Aliquots of 20 μL were then taken at different times (0, 1, 2, 5, 10, and 15 min) and subjected to agarose gel electrophoresis. Cleavage kinetics’ constants (*k_obs_*) were estimated for each complex concentration, taking these reactions as pseudo first-order. The value of *k_obs_* was obtained directly from the angular coefficient of the linear regressions obtained from the plot of the natural logarithm of the intact DNA form quantity versus the action time.

### 3.6. Circular Dichroism

DNA circular dichroism spectra were recorded at different concentrations of the complexes, which were titrated with 200 μM CT-DNA to the ratio of 1:1 [DNA]/[complex], following previously described methods [46].

### 3.7. Fe(II)-EDTA (Hydroxyl radical) DNA Footprinting

In order to determine the capacity and possible preference for specific nucleotide sequences cleavage, a series of assays were performed by replacing the plasmid pBSK II by a synthetic 49-mer oligonucleotide. The oligonucleotide consists of a single strand having a self-complementary region of 42 nucleotides (21 bp) corresponding to two full turns of the double helix and the 5’ end covalently bound to the fluorescent label fluorescein (FAM). We used hydroxyl radical footprinting to detect the site of interaction of the metal complexes with DNA as described by Jain and Tullius [61]. For this assay the DNA was incubated with increasing compound concentrations for 30min, being then added to the reaction: Fe(II)-EDTA (40 mM), sodium ascorbate (40 mM), and H_2_O_2_ (2%) and left to react for 90 s. The reaction was then quenched with 5 μL of thiourea (0.5 M). The protocol for conducting this experiment was the one we have previously described [62]. 

### 3.8. Abl-SH3 Binding Assays

NMR samples of ^15^N-labeled Abl-SH3 domain were prepared following the protocol described by Oliveira, G. A. P. et al. [63]. NMR experiments were carried out at 303 K using Bruker Onebay 400 MHz spectrometer (Billerica, MA, USA) equipped with TXI 5 mm double-resonance probe. The assignment process of the NMR spectra was done using the deposited assignments of ABL-SH3-SH2 in the Biological Magnetic Resonance Data Bank (BMRB; BMRB accession number 4251). NMR spectra were processed using the Topspin 3.2 software (Bruker Biospin S.A, Billerica, MA, USA). The interaction assay between ABL-SH3 and complex **2** was performed using ^1^H-^15^N heteronuclear correlation spectra of the ^15^N-labeled Abl-SH3 domain. The assignment and the ABL-SH3–complex **2** analysis of the titration experiments were done using the software CCPN Analysis. The Chemical Shift Perturbation index (CSP) was calculated using the following equation:

(1) $$\mathrm{CSP}=\sqrt{{\left(\Delta 1H\right)}^{2}+0.15{\left(\Delta 15N\right)}^{2}}$$

where Δ1H and Δ15N are the difference of chemical shifts in the ^1^H and ^15^N dimension on ^1^H-^15^N Sofast-HMQC spectra, respectively. NMR spectra were acquired using samples with 85 μM of ABL-SH3 in phosphate buffer 20 mM pH 7.4 and 5% (*v*/*v*) of dmso-*d6* with and without the addition of at least six times concentrated complex **2**. The dmso-*d6* was added in both samples to improve the solubility of complex 2 in buffed solution. pH was checked before and after the addition of 5% (*v*/*v*) of dmso-*d6* and after the titration of complex **2** to avoid any chemical shift variation due to pH changes. 

The interactions of complexes **1** and **2** with the Abl-SH3 domain were also studied by fluorescence spectroscopy. Protein was dissolved in 20 mM HEPES buffer at pH 7.2. A stock solution of each complex in acetonitrile at the concentration of 1.0 × 10^−2^ M was diluted to 5.0 × 10^−4^ M in HEPES buffer (pH 7.2). To a solution containing 1.0 × 10^−6^ M of protein, increasing concentrations of the tested complexes were added and the emission spectra registered after excitation at 280 nm. Complex concentrations ranged from 0 to 16 × 10^−6^ M. All measurements were performed in triplicate.

### 3.9. BSA Binding Assays

The interactions of complexes **1** and **2** with bovine serum albumin (BSA) were studied by fluorescence spectroscopy. Increasing concentrations of the complexes ranging from 0 to 16 × 10^−6^ M were added to a 1.0 × 10^−6^ M solution of BSA (20 mM HEPES at pH 7.2). The emission spectra were registered after excitation at 280 nm. All measurements were performed in triplicate.

## 4. Conclusions

Two new complexes of ruthenium(II) with mixed ligands, [Ru(bpy)_2_smp](PF_6_) and [Ru(phen)_2_smp](PF_6_), are able to bind to bovine serum albumin and to DNA with a moderate binding affinity. They show preference for thymine base rich sites in DNA. Both compounds are able to inhibit chronic myelogenous leukemia cell growth and the presence of sulfamethoxypyridazine in the metal coordination sphere seems to be crucial for the activity because complexes [Ru(bpy)_2_Cl_2_] and [Ru(phen)_2_Cl_2_] are not active. The substitution of phen for bpy renders the complex more active probably due to the larger extension of the aromatic moiety. Complex **2**, which is the most active, interacts with the SH3 domain of the Abl tyrosine kinase protein, which represents a new cellular target for metal compounds.

## Figures and Tables

**Table 1:** Cytotoxicity of the compounds.

Complex	IC_50_ (μM)
**1**	3.80 ± 0.19
**2**	2.00 ± 0.10
*cis*-[RuCl_2_(bpy)_2_]	>100
*cis*-[RuCl_2_(phen)_2_]	>100

IC_50_ is the concentration required to inhibit 50% of cell growth. The values are the mean of triplicate determinations.

**Table 2:** Kinetic parameters for the reactions catalyzed by complexes **1** and **2**.

	*k_cat_* (h^−1^)	K_M_ (M)	*k_cat_*/K_M_ (h^−1^ M)
**1**	1.38	4.5 × 10^−6^	2.7 × 10^5^
**2**	2.03	3.1 × 10^−6^	6.5 × 10^5^

**Table 3:** Stern-Volmer constants (Ksv), quenching rate constant (kq), binding constant (Kb), number of binding sites (n), and linear regression determination coefficients (R^2^) for the interaction of complexes **1** and **2** with BSA.

Complex	K_sv_ (L mol^−1^)	K_q_ (L mol^−1^ s^−1^)	K_b_ (L mol^−1^)	n	*R* ^2^
**1**	1.02 × 10^5^	1.02 × 10^13^	8.19 × 10^4^	0.99	0.9733
**2**	1.28 × 10^5^	1.28 × 10^13^	2.47 × 10^6^	0.99	0.9793

## Supplementary Materials

The following are available online. Figure S1: Representative scheme of the intermolecular interactions of complex **1**, along z-90, Figure S2: Representative scheme of the intramolecular (top) and intermolecular (bottom) interactions between the molecules of complex **2**, along z-90, Figure S3: The infrared spectrum of complex **1**, Figure S4: The infrared spectrum of complex **2**. Figure S5: Experimental (A) and calculated isotopic distribution (B) for the species [Ru(C_10_H_8_N_2_)_2_(C_11_H_11_N_4_O_3_S)]^1+^, Figure S6: Experimental (A) and calculated isotopic distribution (B) for the species [Ru(C_12_H_8_N_2_)_2_(C_11_H_11_N_4_O_3_S)]^1+^, Figure S7: Photocleavage of supercoiled DNA by **1** and **2** after 5 min of UV-A exposure at 37 °C, pH 7.0, Figure S8: Photocleavage of supercoiled DNA by **1** and **2** after 15 min of UV-A exposure at 37 °C, pH 7.0, Figure S9: Plot of *k_obs_* versus concentrations of complexes 1 (A) and 2 (B). The reactions were performed in HEPES (10 mM) pH 7.0 with increasing concentrations of the complex (5–50 µM), Figure S10: Plasmid DNA cleavage at different pH values ([Buffer] = 10 mM; MES pH 6.0; HEPES pH 7.0, 7.5, and 8.0, and CHES pH 9.0 and 10.0) at 75 µM of 1(A) or 2(B) at room temperature for 4 h, Figure S11: Footprinting experiment in high resolution gel, using an oligonucleotide as substrate, Table S1: Crystal data, data collection, and structure refinement details for [Ru(bpy)_2_smp](PF_6_) and [Ru(phen)_2_smp](PF_6_), Table S2: Selected bond distances (Å) and angles (°) for complex **1** and complex **2**, Table S3: Geometry of hydrogen bonds in complexes **1** and **2**. CCDC reference numbers 1531933 and 1531934 contain the supplementary crystallographic data for complexes **1** and **2**, respectively. These data can be obtained free of charge via www.ccdc.cam.ac.uk/conts/retrieving.html or from the CCDC, 12 Union Road, Cambridge CB2 1EZ, UK; Fax: +44-1223-336033; or e-mail: deposit@ccdc.cam.ac.uk.
