# Supplementary material for: New Heteroleptic Ruthenium(II) Complexes with Sulfamethoxypyridazine and Diimines as Potential Antitumor Agents
Source: Molecules. 2019 Jun 7;24(11):2154. doi: 10.3390/molecules24112154 (PMC6600252; doi:10.3390/molecules24112154)
Supplement: Supplementary file 1 [file molecules-24-02154-s001.pdf]

molecules

Supplementary Material

Article

New Heteroleptic Ruthenium(II) Complexes with Sulfamethoxypyridazine and Diimines as Potential Antitumor Agents

Ariane C. C. de Melo [1], Jaime M. S. V. P. Santana [1], Kelen J. R. C. Nunes [1], Bernardo L. Rodrigues [1], Nathalia Castilho [2], Philipe Gabriel [2], Adolfo H. Moraes [1], Mayra de A. Marques [3], Guilherme A. P. de Oliveira [3], Ívina P. de Souza [1,4], Hernán Terenzi [2] and Elene C. Pereira-Maia [1,*]

[1] Department of Chemistry, Universidade Federal de Minas Gerais, 31270-901 Belo Horizonte, MG, Brazil; ariane.quimica@hotmail.com (A.C.C.d.M.); jaimemurilosvps@gmail.com (J.M.S.V.P.S.); ivina_paula@yahoo.com.br (Í.P.d.S.); cdkelen@hotmail.com (K.J.R.C.N.); bernardo@qui.ufmg.br (B.L.R.); adolfo.dq.ufmg@gmail.com (A.H.M.)

[2] Department of Biochemistry, Universidade Federal de Santa Catarina, 88040900 Florianópolis, SC, Brazil; nathi_zuca@hotmail.com (N.C.); philipe.gabriel.ph@gmail.com (P.G.); hterenzi@ccb.ufsc.br (H.T.)

[3] Programa de Biologia Estrutural, Instituto de Bioquímica Médica Leopoldo de Meis, Instituto Nacional de Biologia Estrutural e Bioimagem, Centro Nacional de Ressonância Magnética Nuclear Jiri Jonas, Universidade Federal do Rio de Janeiro, 21941590 Rio de Janeiro, RJ, Brazil; mayra.marques@ymail.com (M.d.A.M.); gaugusto@bioqmed.ufrj.br (G.A.P.O.)

[4] Department of Chemistry, Centro Federal de Educação Tecnológica de Minas Gerais, 30421-169 Belo Horizonte, MG, Brazil; ivina_paula@yahoo.com.br

* Correspondence: elene@qui.ufmg.br; Tel.: +55-31-3409-5727

Supplementary Figures

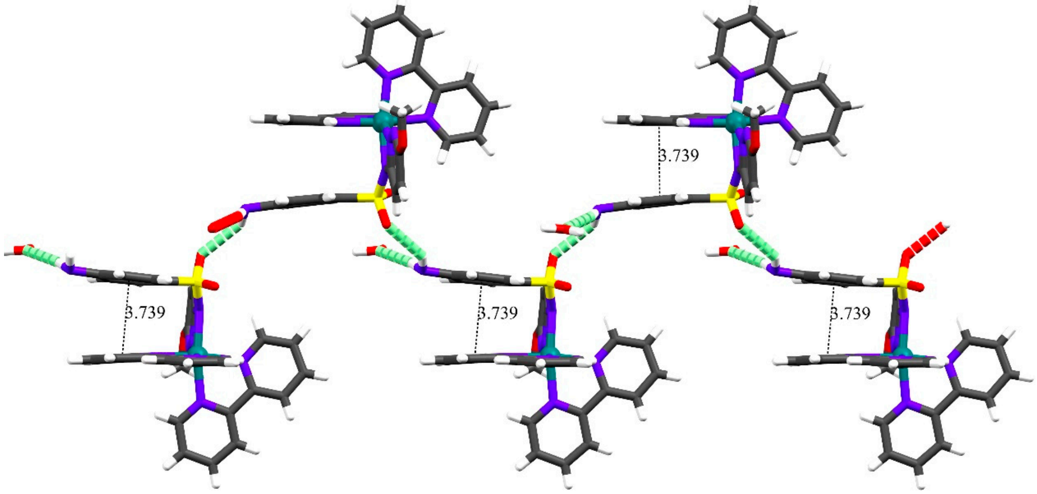

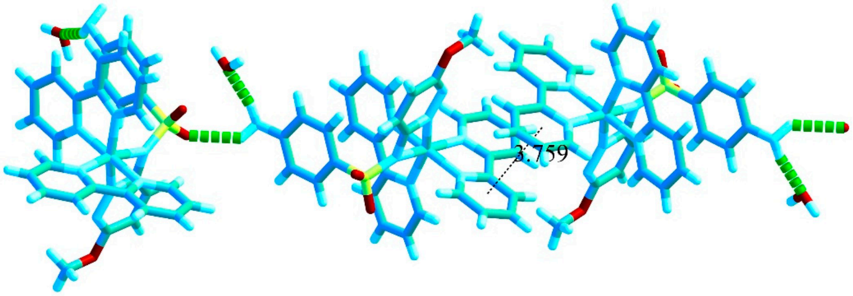

Figure S1. Representative scheme of the intermolecular interactions of complex **1** along z-90. A dashed green line schematizes classical hydrogen interactions between complex **1** and a water molecule. The distances between the centers of bpy and smp or bpy-to-bpy rings are displayed through dashed black lines.

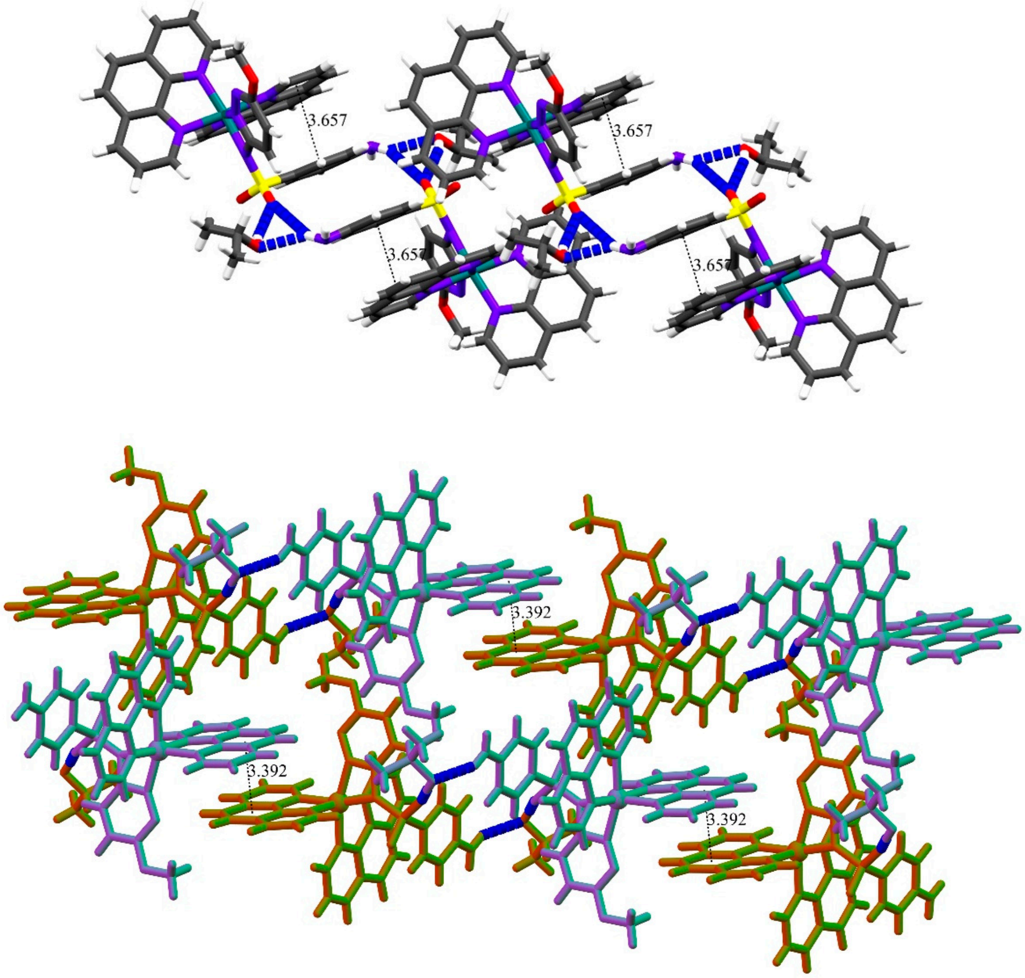

Figure S2. Representative scheme of the intramolecular (top) and intermolecular (bottom) interactions between the molecules of complex **2**, along z-90. Hydrogen interactions between complex **2** and isopropyl alcohol molecule are schematized by dashed blue line. The distances between the centers of phen and smp or phen-to-phen rings are displayed through dashed black lines.

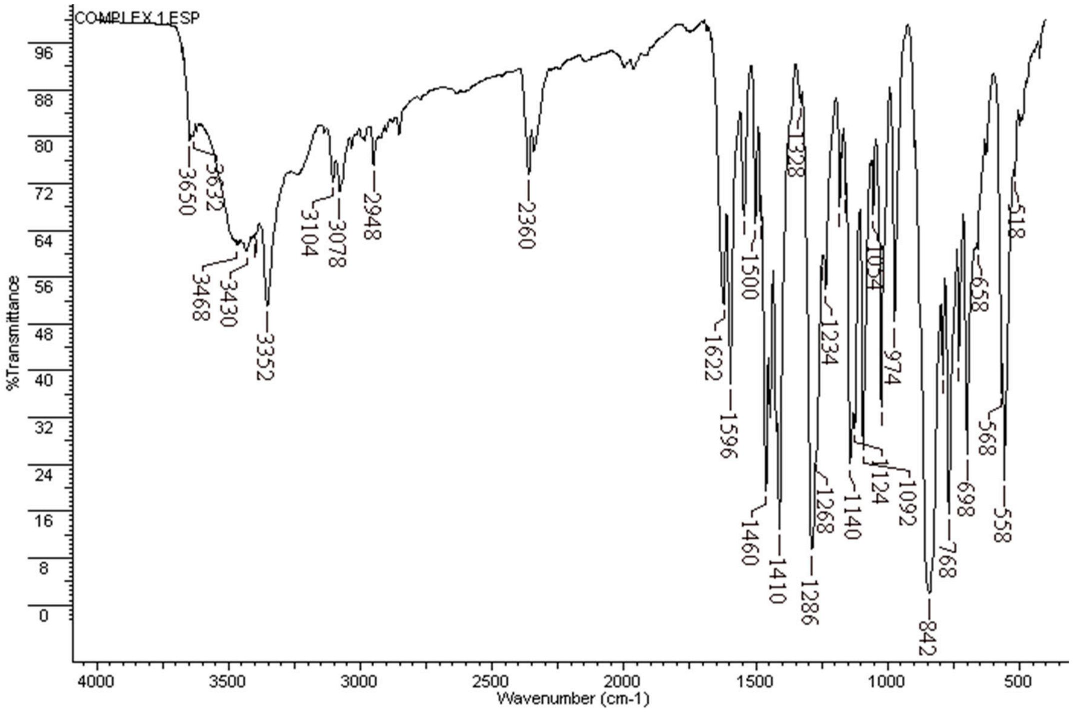

Figure S3. The infrared spectrum of complex **1**.

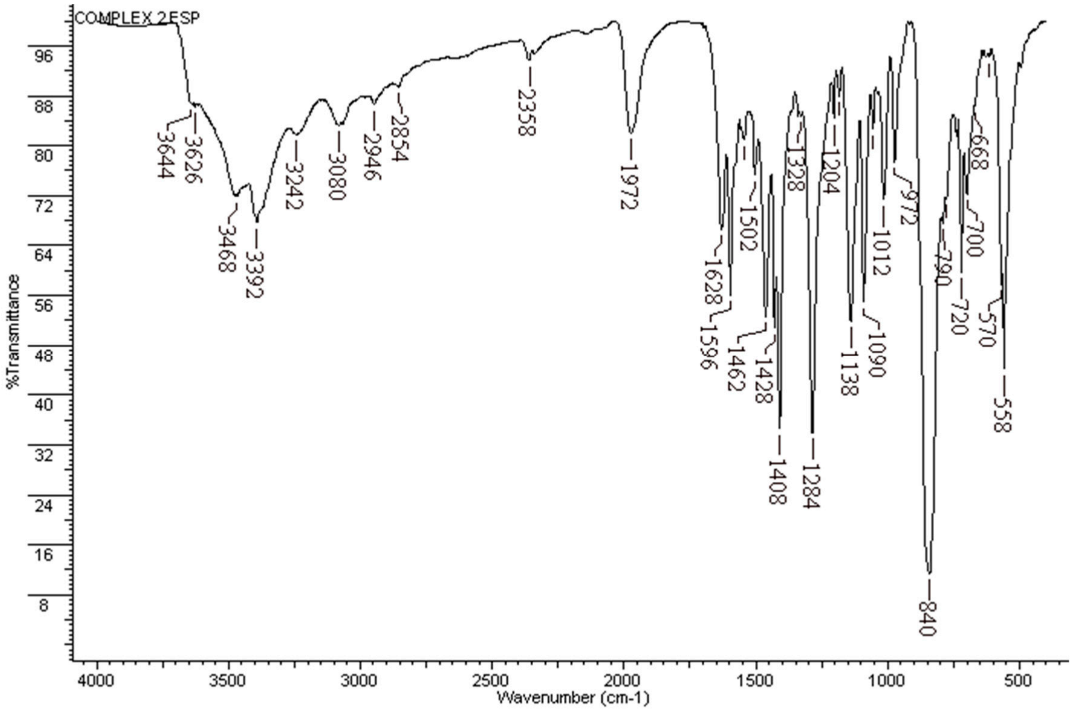

Figure S4. The infrared spectrum of complex **2**.

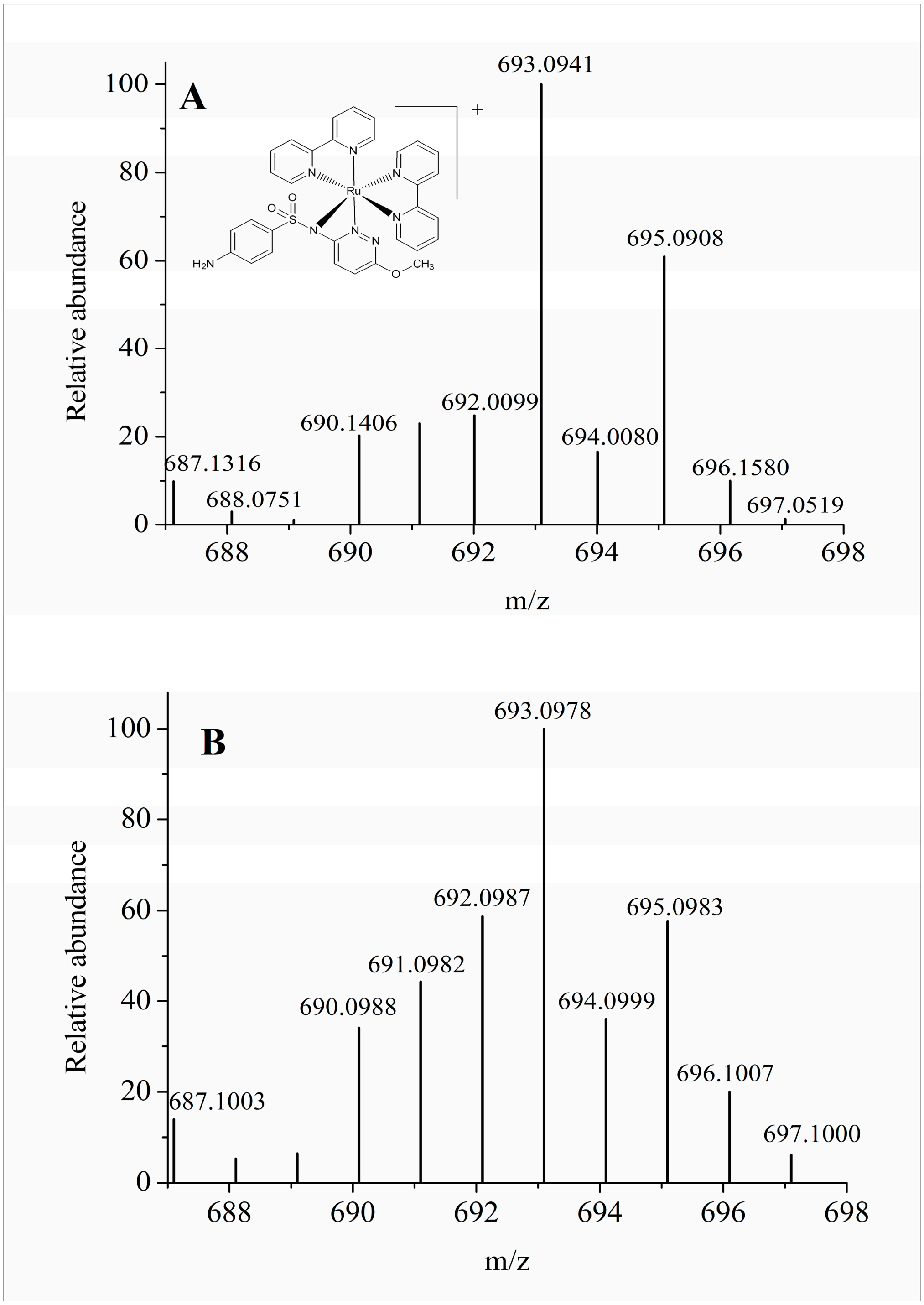

Figure S5. Experimental (A) and calculated isotopic distribution (B) for the species $[Ru(C_{10}H_8N_2)_2(C_{11}H_{11}N_4O_3S)]^{1+}$ in acetonitrile: acetone (1: 1).

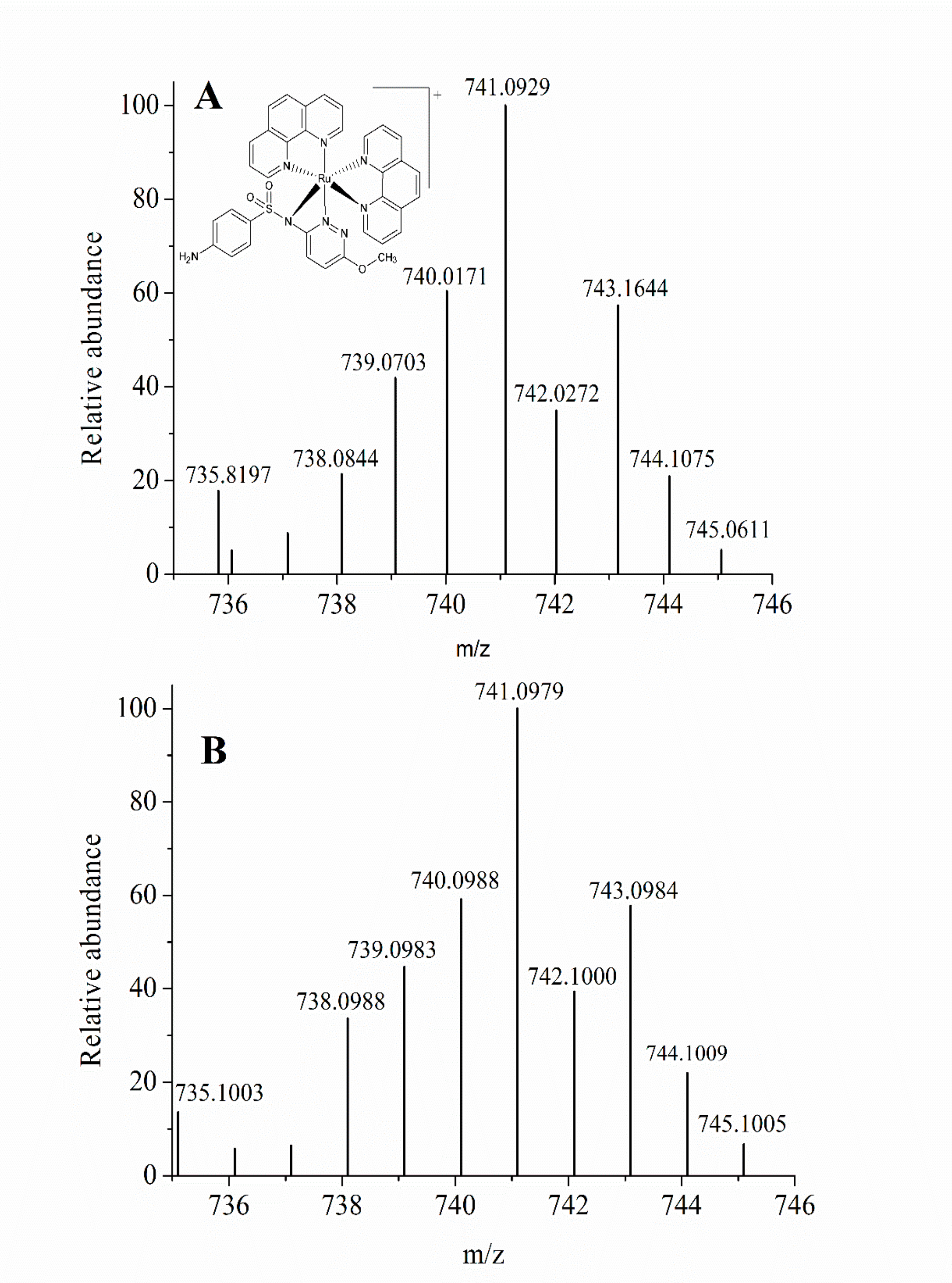

Figure S6. Experimental (A) and calculated isotopic distribution (B) for the species $[Ru(C_{12}H_8N_2)_2(C_{11}H_{11}N_4O_3S)]^{1+}$ in acetonitrile: acetone (1:1).

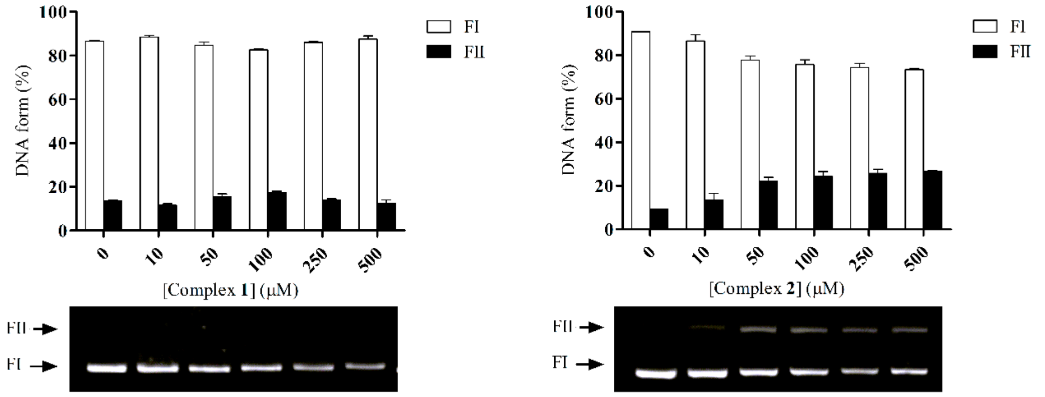

Figure S7. Photocleavage of supercoiled DNA by **1** and **2** after 5 min of UV-A exposure, at 37 °C, pH 7.0.

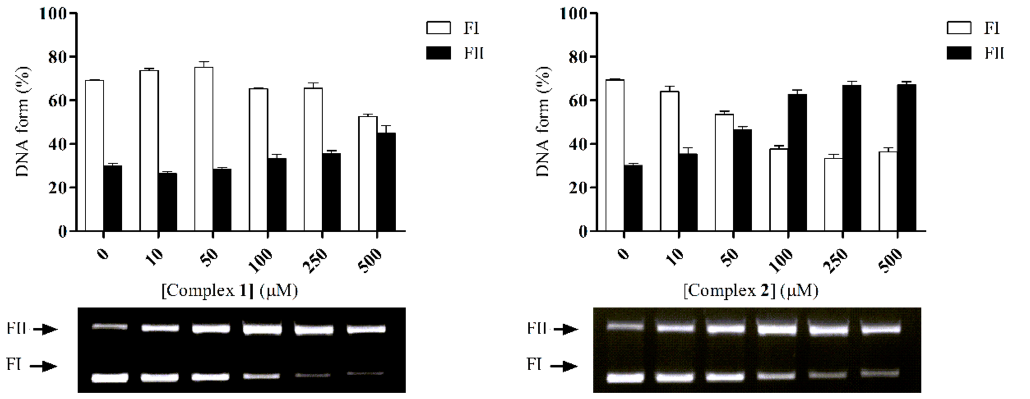

Figure S8. Photocleavage of supercoiled DNA by **1** and **2** after 15 min of UV-A exposure, at 37 °C, pH 7.0.

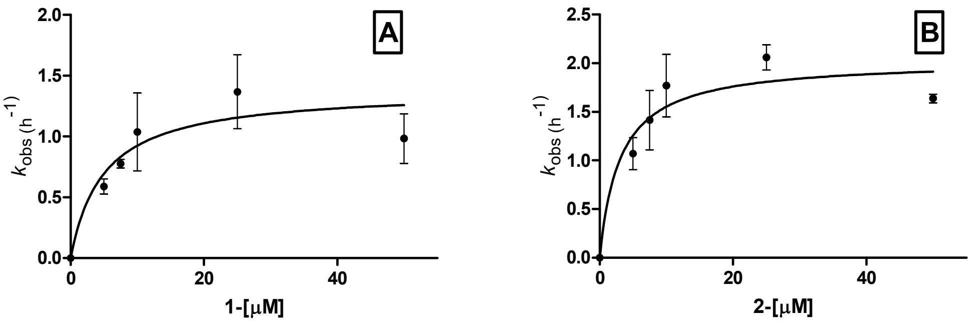

Figure S9. Plot of k_{obs} versus concentrations of complexes 1 (A) and 2 (B). The reactions were performed in HEPES (10mM) pH 7.0 with increasing concentrations of the complex (5 - 50 μM). The incubation was performed in UV light at room temperature with aliquots withdrawn at different intervals of time (0-10min).

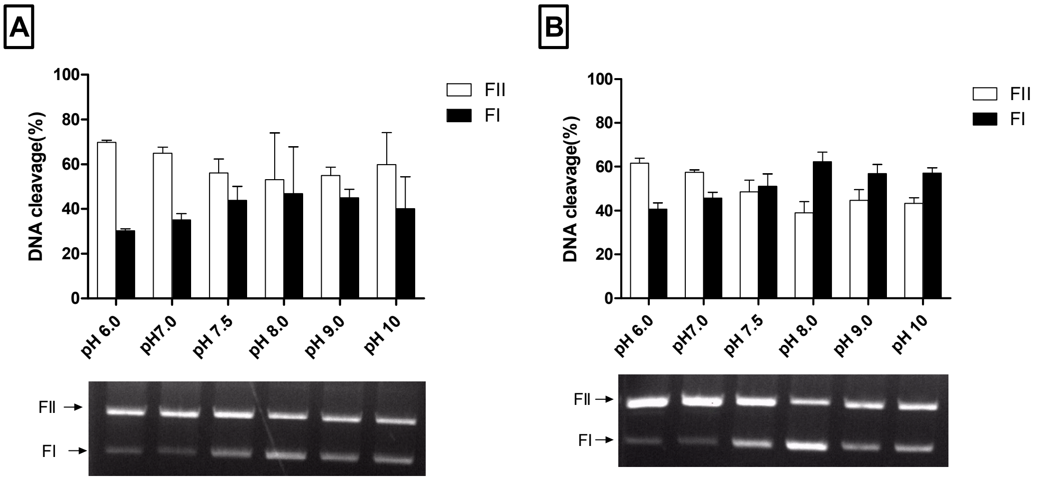

Figure S10. Plasmid DNA cleavage at different pH values ([Buffer] = 10 mM; MES pH 6.0; HEPES pH 7.0, 7.5, and 8.0, and CHES pH 9.0 and 10.0) at 75μM of 1(A) or 2(B), at room temperature for 4h. Representative data from two different tests expressed as mean ± SD.

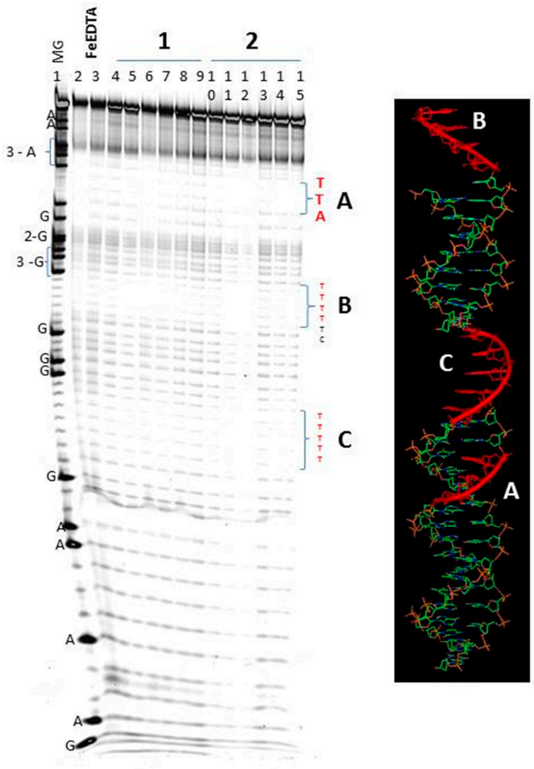

Figure S11. Fe(II)EDTA footprinting experiment on the 42-mer substrate. MG represents Maxam-Gilbert specific cleavage of Adenine + Guanine by formic acid and piperidine treatment. We observe the cleavage promoted by hydroxyl radicals incubated for 60 and 90 seconds (channels 2 and 3) and the protection of this cleavage performed by complex **1** (channels 4 to 9) and **2** (channels 10 to 15). The reactions were conducted for 30 minutes by incubating the oligonucleotide with complexes **1** and **2** for subsequent action of the oxidizing agent for 60 (channels 4, 5, 6, 10, 11, and 12) or 90 (7, 8, 9, 13, 14, and 15) seconds. The oligonucleotide sequence mapping demonstrates protection by complexes **1** and **2** in red regions A, B, and C.

Supplementary Tables

Table S1. Crystal data, data collection, and structure refinement details for [Ru(bpy)$_2$smp](PF$_6$) and [Ru(phen)$_2$smp](PF$_6$).

	**Complex 1**	**Complex 2**
Empirical formula	$C_{31}H_{27.85}F_6N_8O_{3.43}PRuS$	$C_{38}H_{35}F_6N_8O_4PRuS$
Formula weight	845.49	945.84
Temperature/K	120.05	120.45
Crystal system	Monoclinic	Triclinic
Space group	P2$_1$/c	P-1
a/Å	13.2912(4)	13.0077(5)
b/Å	15.9144(3)	13.1547(5)
c/Å	15.8731(4)	14.4323(6)
α/°	90	99.262(3)
β/°	106.929(3)	111.070(4)
γ/°	90	107.141(3)
Volume/Å^3	3212.01(15)	2101.12(18)
Z	4	2
ρ_{calc}g/cm^3	1.7483	1.5314
μ/mm^{-1}	0.689	0.539
F(000)	1702.2	985.7
Radiation	Mo Kα (λ = 0.7107)	Mo Kα (λ = 0.7107)
2Θ range for data collection/°	3.7 to 58.96	3.64 to 59
Index ranges	$-16 \le h \le 16$, $-22 \le k \le 20$, $-21 \le l \le 20$	$-17 \le h \le 17$, $-17 \le k \le 18$, $-19 \le l \le 18$
Reflections collected	29588	45677
Independent reflections	7884 [R_{int} = 0.0299, R_{sigma} = 0.0285]	10566 [R_{int} = 0.0501, R_{sigma} = 0.0422]
Data/restraints/parameters	7884/0/470	10566/0/570
Goodness-of-fit on F^2	1.066	1.048
Final R indexes [I>=2σ (I)]	R_1 = 0.0303, wR_2 = 0.0698	R_1 = 0.0511, wR_2 = 0.1309
Final R indexes [all data]	R_1 = 0.0404, wR_2 = 0.0757	R_1 = 0.0697, wR_2 = 0.1512
Largest diff. peak/hole / e Å^{-3}	0.75/-0.72	1.23/-0.75

Table S2. Selected bond distances (Å) and angles (°) for complex **1** and complex **2**. Standard deviation in parentheses.

**Bond Distances (Å)**			**Bond Angles (°)**		
	**Complex 1**	**Complex 2**		**Complex 1**	**Complex 2**
Ru1–N1	2.0451(16)	2.062(3)	N2–Ru1–N1	79.22(7)	80.18(10)
Ru1–N2	2.0514(16)	2.066(3)	N4–Ru1–N3	79.23(7)	79.88(10)
Ru1–N3	2.0392(18)	2.059(3)	N6–Ru1–N5	62.11(7)	62.20(12)
Ru1–N4	2.0482(16)	2.044(3)	O2–S1–O1	117.35(11)	117.9(2)
Ru1–N5	2.1080(17)	2.131(3)	N5–S1–O1	107.85(10)	107.07(17)

Ru1–N6	2.0982(18)	2.062(3)	N5–S1–O2	109.68(10)	109.09(18)
S1–O1	1.4401(18)	1.436(3)			
S1–O2	1.4448(17)	1.450(3)			
S1–N5	1.5958(18)	1.606(3)			
S1–O1	1.4401(18)	1.436(3)			
S1–O2	1.4448(17)	1.450(3)			

Table S3. Geometry of hydrogen bonds in complexes **1** and **2**. Distances and angles are given in angstroms (Å) and degrees (°), respectively.

D—H---A[a]	D—H	H---A	D---A	D—H---A
**Complex 1**				
N8–H8A---O4[b]	0.928	1.87 8	2.760	157.96°
N8–H8B---O2[c]	0.916	2.324	3.045	135.24°
**Complex 2**				
N8–H8B---O2[d]	0.931	3.000	3.848	152.21°
N8–H8B---O4[e]	0.931	2.226	3.037	145.09°
O4–H4---O2[f]	0.840	2.768	2.768	154.57°

[a] D: hydrogen donor; A: hydrogen acceptor. Symmetry operators: [b] x, y, z; [c] 2- x, -1/2 + y, 1/2 – z; [d] 1 – x, 1 – y, 2 – z; [e] x, y, -1 + z; [f] 1 – x, 1 – y, 1 - z.
